# Autonomic biomarkers of shock in idiopathic systemic capillary leak syndrome

**DOI:** 10.1371/journal.pone.0251775

**Published:** 2021-06-01

**Authors:** Maddalena Alessandra Wu, Emanuele Catena, Antonio Castelli, Roberto Rech, Beatrice Borghi, Davide Ottolina, Tommaso Fossali, Chiara Cogliati, Riccardo Colombo

**Affiliations:** 1 Division of Internal Medicine, ASST Fatebenefratelli Sacco, Luigi Sacco Hospital–Polo Universitario—University of Milan, Milan, Italy; 2 Division of Anesthesiology and Intensive Care, ASST Fatebenefratelli Sacco, Luigi Sacco Hospital–Polo Universitario—University of Milan, Milan, Italy; University of Ljubljana, Medical faculty, SLOVENIA

## Abstract

**Objective:**

The term Idiopathic Systemic Capillary Leak Syndrome (ISCLS) refers to an uncommon condition of severe distributive shock, resulting from an abrupt shift of fluids and proteins from the intravascular to the interstitial compartment. We hypothesise that the autonomic nervous system (ANS) fails in regulating the response to hypovolemia in acute ISCLS and that ANS variables characterise the progression to the recovery.

**Design:**

Prospective cohort study of patients admitted to ICU for severe ISCLS flares.

**Setting:**

Single, referral center in Italy for ISCLS.

**Patients:**

Analysis of cardiovascular signals recorded during seven severe ISCLS attacks and one prodromal period in five patients.

**Interventions:**

ANS was studied non-invasively by means of heart rate variability (HRV) and blood pressure variability analysis, as an estimation of vagal and sympathetic modulation directed to the heart and vessels. Heart rate and systolic arterial pressure (SAP) variability were also used to assess baroreflex sensitivity. ANS variables were measured during the subsequent phases which characterise ISCLS flares, namely the acute phase, the post-acute phase, and the recovery phase.

**Measurements and main results:**

HRV was severely depressed during the acute phase accounting for the loss of ANS modulation during massive capillary extravasation. This phase was characterised by shock and impaired baroreflex control, which allowed SAP to oscillate driven by respiratory activity. Impending shock and transition from shock to a post-acute phase were marked by change of baroreflex spectral variables. The baroreflex control was fully restored during recovery.

**Conclusions:**

ANS modulation and baroreflex control are severely impaired during the acute haemodynamic instability which characterises ISCLS crises and their progressive restoration may be a clue of improvement. ANS indices during ISCLS flares might serve as useful biomarkers, able to timely announce the transition from one phase to the subsequent one, thus helping to adapt therapy accordingly.

## Introduction

Idiopathic Systemic Capillary Leak Syndrome (ISCLS) is a rare disease of unknown aetiology which presents with acute, recurrent and often fatal episodes of hypovolemic shock [1]. The diagnosis relies on the triad of unexplained shock, severe haemoconcentration and hypoproteinemia [2]. Due to the rarity of the disease (about 300 cases described since 1960), the pathological mechanisms are mostly unknown, and therapy is still controversial.

After a short prodromal period, the attack is usually characterised by an abrupt onset. Plasma extravasation leads to arterial hypotension and even life-threatening shock, characteristically unresponsive to fluids and catecholamines infusion (acute phase). If the patient survives this stroke, the intravascular volume is then progressively restored (post-acute phase) until full recovery (recovery phase) [3, 4].

Mild upper airway infections often precede the attacks; thus, the hypothesis of an immune-mediated origin of ISCLS has been extensively investigated. Several alterations in cytokine pathways and cell-mediated immune responses have been alternately hypothesised to have a role [5–8]. However, the mechanisms underlying the clinical picture are still only partially understood and involve a loosening of endothelial junctions, causing a sudden increase of capillary permeability to proteins and leading to a protein-rich fluid shift from the intravascular to the interstitial space. Therefore, from the laboratory point of view, the main features of the acute phase are severe haemoconcentration and hypoalbuminemia. As a result of the poor awareness of ISCLS and due to the clinical picture of the crisis (surge, unresponsive hypotension, and marked leukocytosis due to haemoconcentration), ISCLS is usually misdiagnosed as septic shock, and its true incidence is most likely underestimated.

The endothelial lesion is presumably functional because it resolves within 24–72 hours [9, 10]. Clues suggesting recovering from the crisis include more stable haemodynamic parameters, often passage from oliguria to polyuria (even though this may occur later), as well as the change in the trend of haematocrit values which become stable and afterwards decrease towards baseline values. Serum protein concentrations usually require more time (even a couple of weeks) to revert to normal. Overlapping cardiogenic shock due to myocardial edema and acid-base balance derangement further complicate the clinical picture of the attacks [11, 12]. Complications developed during the shock, sometimes persist during recovery and may even become long-lasting, contributing significantly to ISCLS morbidity [3, 13].

The autonomic nervous system (ANS) usually compensates for the state of hypovolemia by increasing the firing rate of sympathetic fibres and decreasing the vagal outflow aiming at maintaining cardiac output and organ perfusion. However, ANS activity is frequently deranged in critically ill patients, and its dysfunction has been correlated with poor outcome [14–16]. Furthermore, the ANS may have a role in the regulation of the inflammatory pathway reflex through the so-called “cholinergic anti-inflammatory pathway” [17, 18]. Unfortunately, data from studies in a general Intensive Care Unit (ICU) population suffer from two significant confounders: deep sedation and mechanical ventilation, both of which are used in almost all critically ill patients. With these premises, it is difficult to draw general conclusions in this setting, but hints that hemodynamics, ventilation and inflammation have a strict interplay with the ANS network are appealing.

We previously reported that heart rate variability was markedly depressed during the shock phase of ISCLS crisis, potentially contributing to ANS failure and accounting for the severity of shock [19]. This study aimed at evaluating the hypothesis that indices of autonomic nervous system modulation may help to discriminate the subsequent phases which characterise life-threatening ISCLS flares, namely the acute, the post-acute and the recovery phase.

## Materials and methods

The Ethics Committee "Comitato Etico di Area 1, Milan" approved the publication of the collected data in an anonymized form and the patients provided their written consent. From April 2014 to April 2019, we prospectively collected data from five patients, who were consecutively admitted to our ICU at the Luigi Sacco Hospital in Milan, a university referral centre for ISCLS in Italy, because of nine episodes of life-threatening ISCLS attack. Data from one more case admission, occurred during the prodromal period, were analysed and discussed separately.

We considered the three phases of the course of ISCLS crisis according to the following criteria: i) the *acute* phase, from ICU admission for refractory shock until peak hematocrit values; ii) the *post-acute* phase, from the beginning of hematocrit reduction and progressive increase of arterial pressure until iii) *recovery* phase, characterised by mean arterial pressure ≥65 mmHg, heart rate <100 bpm, lactate ≤2 mmol∙L^-1^, without the need of mechanical circulatory support, vasopressors or inotropes infusion, and diuresis >0.5 ml∙kg∙hr^-1^. The crises were managed conservatively according to the previously published algorithm [3].

### Heart rate and systolic arterial pressure variability analysis

Autonomic nervous system modulation on the cardiovascular system was assessed by means of heart rate and systolic arterial pressure (SAP) variability analysis. Cardiovascular signals were collected with patients lying supine, avoiding administration of fluid challenge during data recording. ECG and invasive blood pressure were collected simultaneously for at least 10 min through the intensive care unit multiparametric monitor Philips IntelliVue MX800 (Philips Healthcare, Amsterdam, The Nederland) on the built-in PC running iXtrend software (ixellence, Wildau, Germany) and sampled at 500 Hz and 125 Hz respectively. All signals were eventually resampled at 500 Hz with Labchart Pro 8 (ADInstruments, Dunedin, New Zealand). Heart rate and SAP variability were analysed offline with HeartScope II (AMPS LLC, New York, USA). The only patient who was admitted to ICU during the prodromal phase underwent HRV and SAP recordings within 24 hours from ICU admission as well. ECG and invasive blood pressure were recorded while he was lying calmly supine and during a modified tilt manoeuvre (MTILT), as previously described [20]. His data are shown separately from the other cases admitted to ICU because of circulatory shock.

After detecting the QRS complex on the ECG, the temporal distance between two consecutive R peaks (R-R interval) was computed and used as a measure of the heart period. The maximum of arterial pressure inside each heart period was taken as SAP. The recognition of R and SAP peaks were manually checked by an investigator to avoid erroneous detections or missed beats. If isolated ectopic beats affected heart period and SAP values, these measures were linearly interpolated using the closest values unaffected by ectopic beats. The series were discharged if the ectopic beats were >5% of the sinus beats, and a new window of the series was then considered. Sequences of 500 beats were analysed. Respiration series were obtained from the respiratory-related amplitude modulation of the ECG.

The power spectrum was estimated by means of a univariate parametric approach fitting the series according to an autoregressive model [21]. Both heart period and SAP autoregressive spectral densities were factorised into two components: low frequency (LF) if the central frequency was between 0.04 and 0.15 Hz, and high frequency (HF) if the central frequency was between 0.15 and 0.5 Hz [22]. Thus, in this paper, we used the labels HF_RR_ and HF_SAP_ to indicate the spectral power of the heart period and the SAP in the high frequency band, respectively. Similarly, we used the label LF_RR_ and LF_SAP_ to indicate the spectral power of the heart period and the SAP in the low frequency band, respectively. The HF_RR_ was used as a marker of vagal modulation directed to the heart, while the LF_SAP_ was used as a marker of sympathetic modulation directed to the vessels [22–24]. Moreover, LF_RR_ reflects vasomotor activity, which is an indirect index of sympathetic nerve activity but is also affected by parasympathetic activity [25–27]. The HF_SAP_ does not directly reflect autonomic nervous system modulation, and we considered it a measure of the magnitude of arterial pressure oscillation in the high frequency band. The amplitude of both LF and HF components is assessed by the area (i.e. power) of each component and, therefore, squared units are used for its absolute value: squared milliseconds (msec^2^) for the heart period and squared millimetres of mercury for the SAP (mmHg^2^).

The frequency domain analysis of HRV by the autoregressive method is a powerful tool to quantifying the contribution of each component (LF and HF) to the spectrum, and it is more noise independent than other methods (i.e. Fast Fourier Transform) especially in the high frequency bands. Unfortunately, the autoregressive analysis has the prerequisite of the signal stationarity. Thus, it is not an optimal method to detect fast-fluctuating phenomena and transients. For this purpose, we used a wavelets analysis approach to assess the ongoing ANS response to a gravitational challenge in the patient who underwent modified tilt. For this item, we used HRVanalysis 1.1 for Windows (ANSlabtools, Saint-Ètienne University) [28].

### Baroreflex assessment

The baroreflex control was calculated with different approaches. The powers of heart period and SAP series in LF and HF bands (LF_RR_, HF_RR_, LF_SAP_ and HF_SAP_) were used for the estimation of the baroreflex control according to an autoregressive spectral approach and were expressed as α_LF_ and α_HF_ for the low frequency and high frequency bands, respectively.

Furthermore, the baroreflex sensitivity (BRS) was calculated with the sequence method (sequence criteria: correlation coefficient > 0.85, sequence length = 4 beats, delta SAP > 1 mmHg, delta RR > 5 msec) [29]. Moreover, baroreflex sensitivity was also calculated with an open-loop causal double exogenous autoregressive model and labelled as αXXAR for the study purpose [30].

### Haemodynamic parameters and blood samples

According to the usual clinical practice, hemodynamic data, as well as blood sample analyses and parameters of acid-base status, were recorded during the ICU stay: (i) within 12 hours from patient admission for the acute phase, (ii) within 12 hours from the hematocrit reduction for the post-acute phase, and (iii) within 12 hours from the hematocrit stabilisation and arterial pressure return to the usual values in each patient for the recovery phase. Furthermore, haemodynamic data were recorded within 12 hours from ICU admission in the patient admitted during the prodromal period.

### Statistics

Data are shown as mean (range). The normality of the distribution of measured variables was checked with Kolmogorov-Smirnov normality test. Multiple comparisons were analysed with one-way ANOVA followed by Bonferroni’s post hoc test or one-way ANOVA on ranks followed by Dunn’s post hoc test when appropriate. Comparison between categorical variables was performed by the Fisher’s exact test. Two-tail p value <0.05 was considered statistically significant for all tests. Sigmaplot 11 (Systat Software, San Jose, CA, USA) was used for the statistical analysis.

## Results

Five patients, with a total of seven severe ISCLS attacks, were considered for data analysis. They were all male, mean age 46.9 years (range 36–60). The mean ICU stay was 4.5 days [3–9]. Furthermore, a patient, with a history of ISCLS, was admitted to ICU during the prodromal period, which was characterised by orthostatic hypotension, limb swelling, and oliguria following an episode of upper airway infection. This subject did not develop shock afterwards and his data are described separately.

In two episodes, the patients were sedated and mechanically ventilated, in one of them the patient underwent extracorporeal circulatory support because of severe myocardial oedema causing mixed, hypovolemic and cardiogenic, shock. All other episodes were managed conservatively with the maintenance of spontaneous breathing, restrictive use of fluids, permissive hypotension, avoiding sedation, and tolerating anuria [3].

In two ISCLS episodes, the cardiovascular signals were not stored; thus, HRV analysis was performed on signals recorded in seven severe episodes during the ICU stay. In one case, only the electrocardiogram (ECG) recorded during the first day was available, and the blood pressure signal was discarded because the patient was on extracorporeal circulatory support by venous-arterial ECMO. In one episode, only two recordings were available (during acute and post-acute phase respectively). In another episode, only recordings during the post-acute and recovery phase were available because the patient was initially managed in his county hospital and transferred to our ICU on the second day when the picture of shock was improving.

The haemodynamic and laboratory results of patients admitted because of life-threatening crises are shown in Table 1.

**Table 1:** Haemodynamic and laboratory analyses of ISCLS patients admitted to ICU because of shock.

Variable	Acute phase	Post-acute phase	Recovery phase	p
**Haemodynamics**				
HR, mean (range), bpm	125.7 (94.5–146.9)	96.8 (90–105)*	74 (67.7–85.8)*	<0.001
SAP, mean (range), mmHg	75.4 (55–96.1)	109.2 (94.9–119.9)*	122.6 (102.7–149)*	<0.001
DAP, mean (range), mmHg	50.7 (40–60.8)	70 (60.3–77.4)*	72.6 (56.3–90.5)*	0.008
**Blood analysis**				
Hb, mean (range), g/dL	19.8 (12.1–24.7)	16.8 (9.8–20.1)	10.6 (7.7–13.4)*§	0.002
Ht, mean (range), %	57.2 (35.3–72)	48.9 (27.9–57)	31.1 (23–40)*§	0.002
RBC, mean (range), 10^3^ cells/mm^3^	6815 (4090–7990)	5845 (3510–7070)	3675 (2750–4700)*§	0.002
WBC, mean (range), cells/mm^3^	38878 (18520–66440)	23817 (6250–42470)	10300 (7520–13840)*	0.009
PLT, mean (range), 10^3^ platelets/mm^3^	223.6 (164–294)	254.5 (159–353)	173.1 (94–336)	0.14
PTT, mean (range), INR	1.56 (0.97–2.36)	1.2 (0.9–1.6)	1.02 (0.77–1.15)*	0.03
PT, mean (range), INR	1.56 (1.19–2.16)	1.22 (1.1–1.44)	1.1 (1.04–1.2)*	0.08
D-dimer, mean (range), μg/L	1354 (503–3609)	1243 (789–1770)	827 (388–1065)	0.64
Urea, mean (range), mg/dL	86 (67–116)	144 (86–303)	99.8 (36–172)	0.3
Creatinine, mean (range), mg/dL	3.18 (2.2–4.3)	3.8 (1.9–5.7)	2.1 (1.03–4.3)	0.06
Albumin, mean (range), g/dL	1.9 (0.9–2.5)	2.8 (2.1–3.5)	3.2 (2.7–3.7)*	0.008
C-reactive protein, mean (range), mg/L	43 (7–88)	44.5 (17–111)	35.3 (8.9–67)	0.86
CPK, mean (range), U/L	285 (129–540)	1806 (118–9084)	113 (28–206)	0.1
Lactate, mean (range), mmol/L	4.2 (2.2–8.1)	1.8 (0.9–2.8)	0.8 (0.5–1)*	0.003

Haemodynamic data, blood sample analyses and acid-base status of patients admitted to ICU because of life-threatening ISCLS attacks. HR, heart rate; SAP, systolic arterial pressure; DAP, diastolic arterial pressure. Values are shown as mean (range).

*p<0.05 with respect to the acute phase.

§p<0.05 with respect to the post-acute phase.

**Fig 1:**
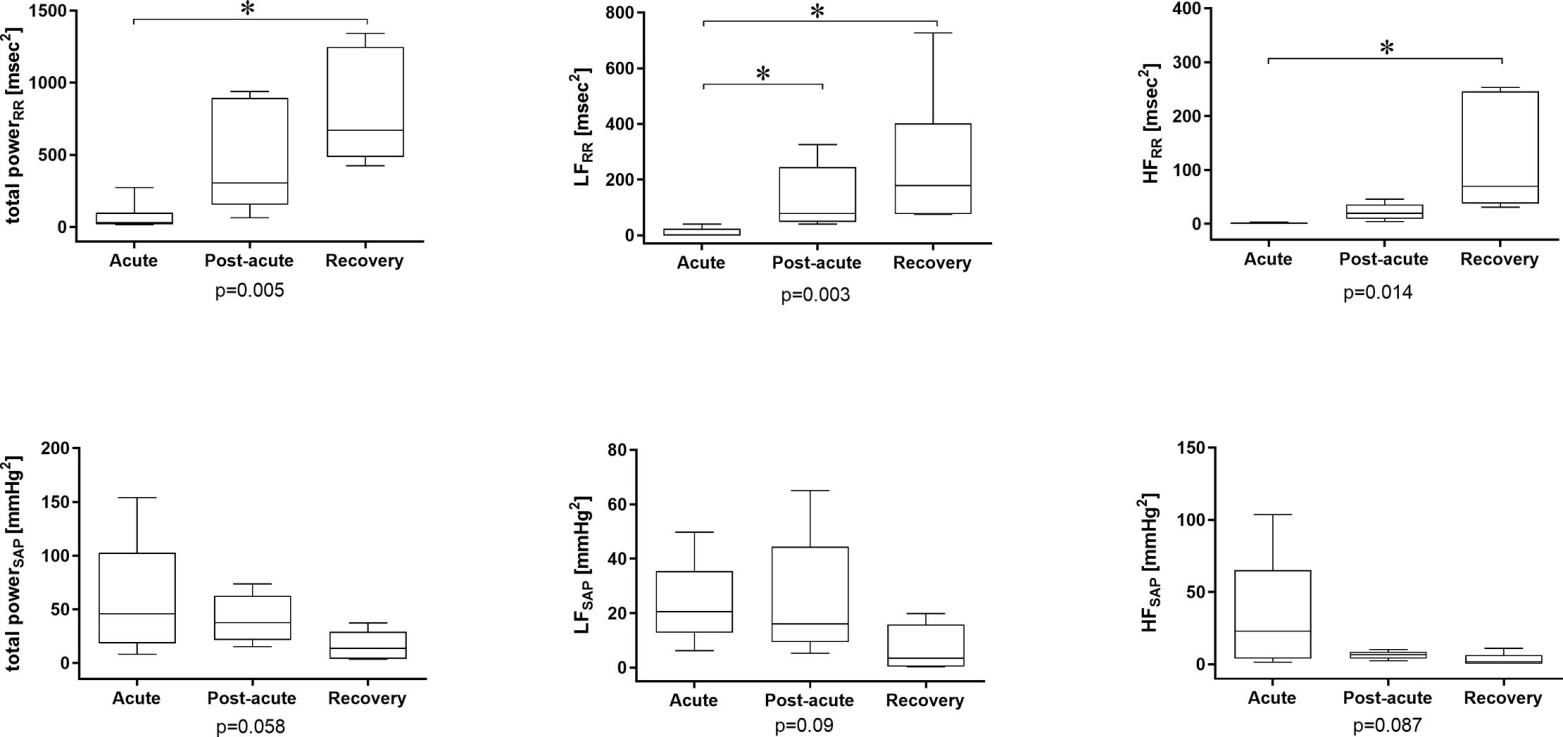
Heart Rate (RR) and Systolic Arterial Pressure (SAP) variability analysis results in the frequency domain. RR, heart period; SAP, systolic arterial pressure; HF, high frequency band of the power spectrum; LF, low frequency band of the power spectrum. *p<0.05 for post-hoc analysis of ANOVA on ranks.

The cardiovascular findings of heart rate and SAP variability analysis are shown in Fig 1. During the acute phase, all patients showed an extremely low overall heart rate variability, which increased during the post-acute phase and was regained at the recovery phase (p = 0.017). The vagal modulation was severely depressed during the acute phase and gradually reappeared in the following phases (p<0.01).

**Fig 2:**
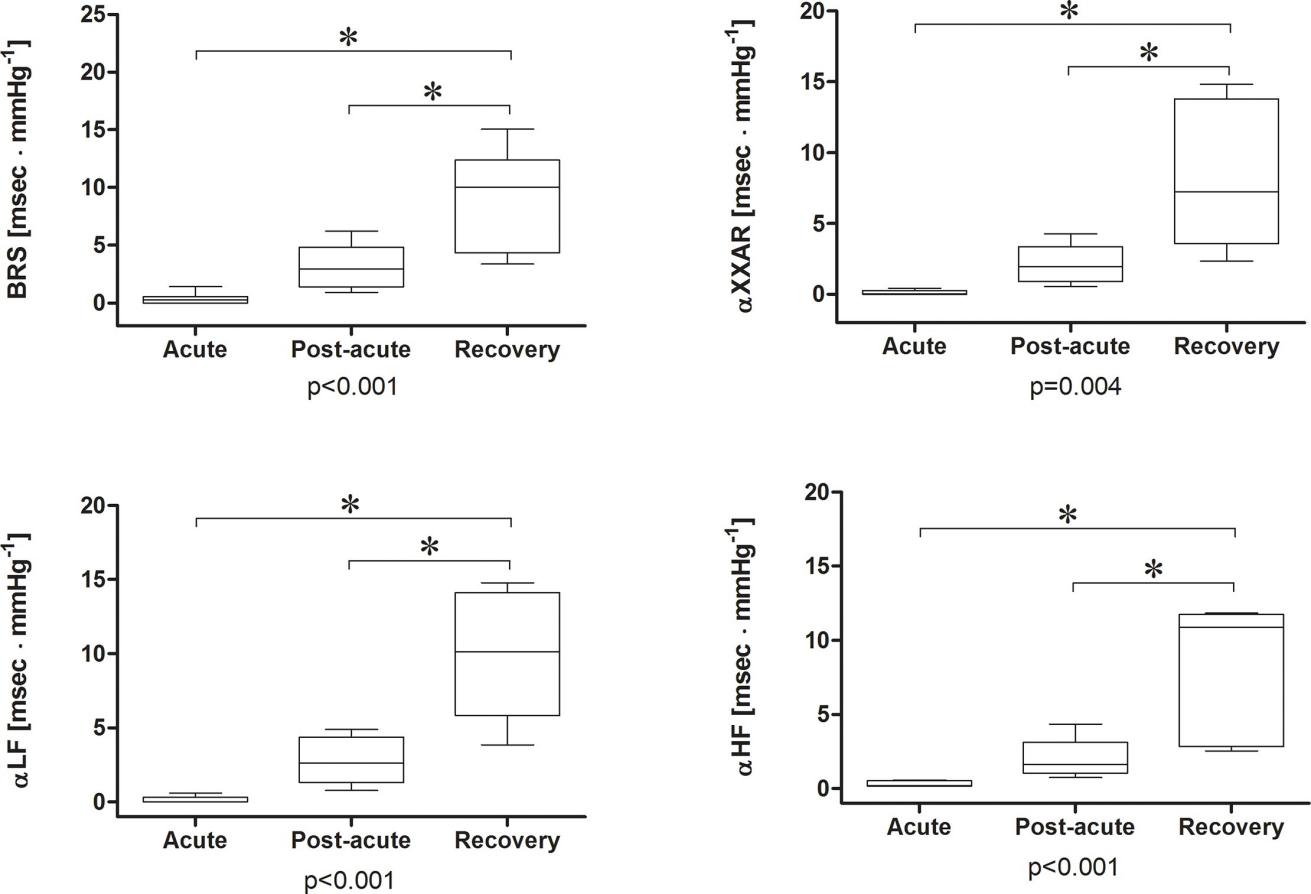
Baroreflex control of patients who experienced shock during idiopathic systemic capillary leak syndrome flares. The baroreflex control was severely depressed during the shock phase and gradually reappeared during the post-acute and recovery phases. *p<0.05 for post-hoc analysis of ANOVA on ranks.

The baroreflex control was severely depressed during the acute phase and gradually reappeared during the post-acute and recovery phases (Fig 2). The SAP showed maximal variability in the acute phase [56 (8–154) mmHg^2^], especially in the high frequency band [32 (1–103) mmHg^2^].

The acute phase, compared to the following phases, was characterized by a total power density of the heart period <50 msec^2^ (OR 84.3, 95%CI 2.9 to 2425, p = 0.001), LF_RR_ <50 msec^2^ and/or HF_RR_ <10 msec^2^ (82.2, 95%CI 2.9 to 2355, p<0.001), BRS <1 msec∙mmHg^-1^ (OR 50, 95%CI 2.6 to 977.7, p = 0.005), αLF <1 msec∙mmHg^-1^ (OR 91, 95%CI 3.2 to 2587, p<0.001), αHF <1 msec∙mmHg^-1^ (OR 91, 95%CI 3.2 to 2587, p<0.001), and αXXAR <1 msec∙mmHg^-1^ (OR 91, 95%CI 3.2 to 2587, p<0.001).

**Fig 3:**
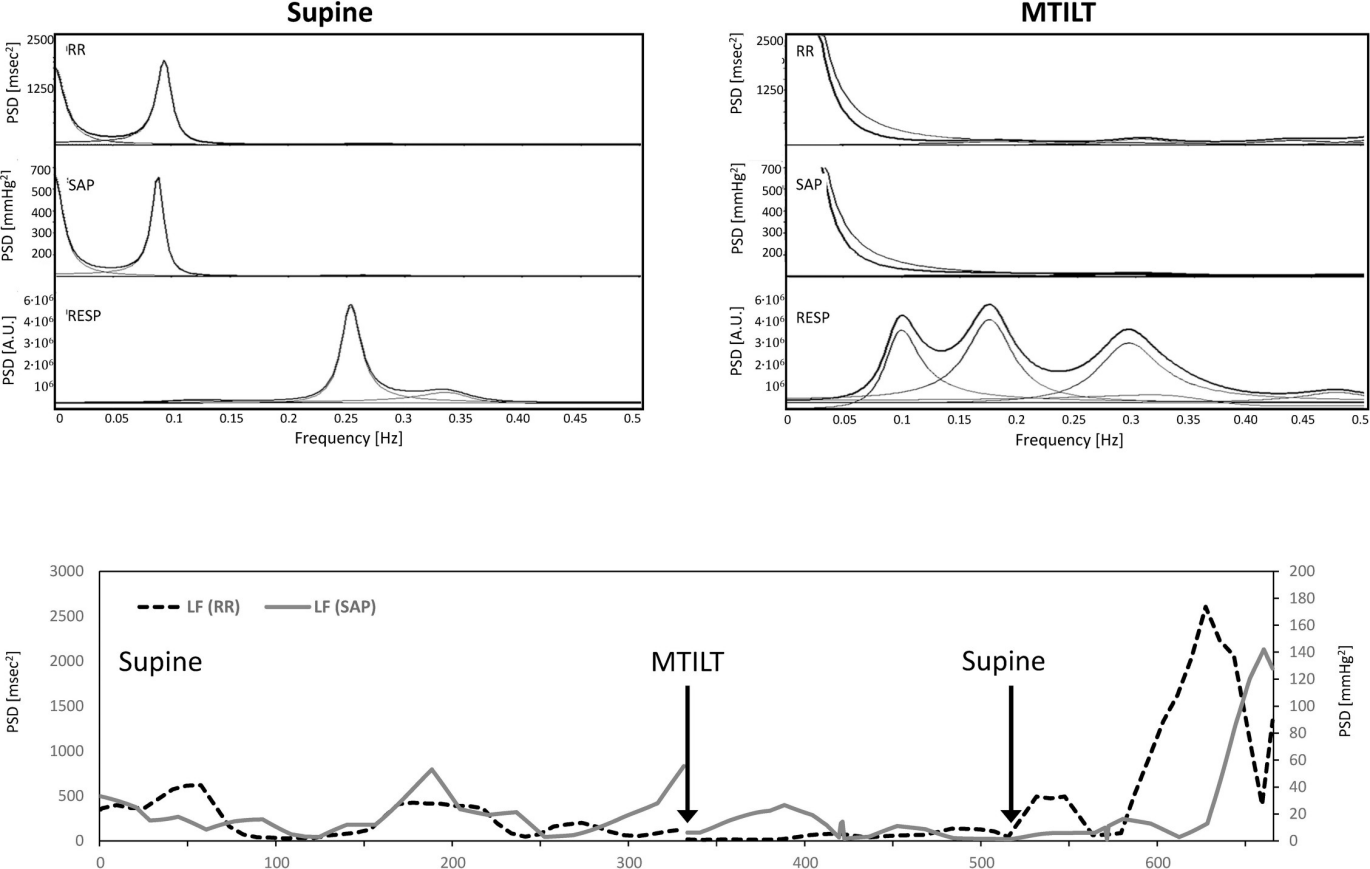
Graphical representation of heart period and systolic pressure variability of a patient admitted to ICU during the prodromal phase and passive modified tilt (MTILT). At the top, the power spectra derived from 300 beats at baseline and during MTILT are shown. MTILT induced a marked reduction of LF spectral densities of heart period and SAP. At the bottom, the pattern of LF spectral densities of heart period and SAP changes assessed by wavelets analysis is shown. Both LF densities reappeared after the MTILT interruption. *PSD*, power spectral density.

The patient who was admitted during the prodromal period did not develop shock. The haematocrit increased from 38% to 49%, and urine output was low (0.3 ml∙kg∙hr^-1^ in the first 24 hours). The arterial blood pressure remained within his usual values. He was managed with complete rest and reduction of fluid intake. He was discharged to medical ward on the third day. The spectral analysis of heart rate and systolic pressure variability at rest showed a predominant sympathetic modulation without vagal activity. During MTILT the blood pressure quickly dropped, and the patient experienced visual loss, cold diaphoresis, profound malaise, agitation, and confusion (pre-syncope). The maneuver was aborted, and symptoms rapidly resolved. The sympathetic modulation directed to the vessels (LF_SAP_) disappeared (Fig 3). The baroreflex control was also affected by MTILT with respect to the supine position: BRS was 1.2 *vs*. 2.04 msec∙mmHg^-1^, αLF 0 *vs*. 2.04 msec∙mmHg^-1^, αHF 2.03 *vs*. 1.61 msec∙mmHg^-1^ and αXXAR 0.35 *vs*. 1.01 msec∙mmHg^-1^. On the second day, the patient was asymptomatic and was able to tolerate MTILT without symptoms. At that time, during MTILT, BRS was 1.97 *vs*. 1.5 msec∙mmHg^-1^ at baseline, αLF 1.93 *vs*. 1.58 msec∙mmHg^-1^, αHF to 0.96 *vs*. 0.89 msec∙mmHg^-1^, αXXAR 1.64 *vs*. 1.82 msec∙mmHg^-1^. The spectral densities of LF_RR_ increased during MTILT to 154 *vs*. 45 msec^2^ and LF_SAP_ to 41.5 *vs*. 18 mmHg^2^ from baseline.

All patients were discharged alive to home from hospital.

## Discussion

Our findings account for a profound and unexpected derangement of ANS modulation in the course of life-threatening episodes of ISCLS. Also, HRV indices, including some baroreflex sensitivity variables, may differentiate the acute, post-acute and recovery phase. In fact, HRV variables characterise each phase during ISCLS flares. Furthermore, ANS analysis might be meaningful to give hints to the transition from the acute phase to the post-acute phase.

In physiological conditions, a hypotensive stimulus, such as hypovolemia, induces an ANS response increasing sympathetic and lowering vagal modulation, which aims at maintaining the central blood volume and perfusion pressure within normal ranges. In this ISCLS series, despite the severe shock, there was a drop in the overall heart rate variability during the acute phase. Similarly, such an impairment of autonomic nervous system modulation on the cardiovascular activity was observed only in patients under deep anaesthesia in the absence of surgical stimuli or with denervated hearts after transplantation [31, 32]. In our patients, the heart rate variability gradually increased throughout ICU stay with a progressive increase of both sympathetic and vagal components.

As mentioned in the introduction, the course of ISCLS attacks is divided into acute, post-acute and recovery phases. The crisis is preceded by a prodromal period, usually developing outside the hospital. Our data suggest that the life-threatening crises needing ICU admission are characterised by abrupt downregulation of autonomic modulation of haemodynamics and baroreflex control during the acute phase, which progressively was restored with the improvement of the clinical condition. The haematocrit reduction, in the absence of blood loss, suggests that endothelial junctions are closing and that passage of fluids from the extra to the intravascular space is beginning. The transition from the acute to the post-acute phase is a crucial turning point which should be identified timely to modify the therapeutic approach accordingly. During the acute phase, the endothelial tight junctions are open; thus, most of the administered fluids freely cross the endothelial barrier. At that time, fluid resuscitation worsens the microcirculatory perfusion because it increases interstitial oedema without improving the shock picture, and the patients remain fluid unresponsive. When the tight junctions close, administered fluids and colloids are more prone to stay into the intravascular compartment (beginning of fluid-responsiveness). Our results show that the transition from the acute to the post-acute phase is characterised by specific autonomic markers. Remarkably, acquiring signals which might be suitable for HRV analysis and for evaluation of the baroreflex in humans in other kinds of shock (e.g. septic or hemorrhagic shock) can be extremely difficult, subject to many possible confounding factors (e.g. due to the impact of amines or of deep sedation-anesthesia on the sympatho-vagal balance) and the intrinsic wide heterogeneity among patients may limit reproducibility, comparability and generalizability of results. Moreover, most of the studies published so far, even in animal models providing experimental conditions which are not affected by possible “real life” confounders, were either not able to discriminate among phases following the acute shock [33] or tried to unravel alterations in the modulation of ANS parameters during subsequent phases but highlighted results which differ significantly from ours [34]. On the opposite, the possibility to discriminate among ISCLS phases thanks to changing HRV indices is one of the undeniable strengths of our results. Therefore, our findings should be considered valuable and might be useful to assist clinicians in the management of ISCLS flares, guiding judicious and timely use of fluids as well as other therapeutic interventions.

The early admission to ICU of a patient during the prodromal period provided us with the unique opportunity to study ANS modulation during the period that usually precedes shock. Patient’s ANS was stimulated by a modified tilt manoeuvre, which in healthy subjects elicits a sympathetic response. Interestingly, in this subject, the ANS failed to produce a sympathetic activation in response to the gravitational challenge, which appeared after the return to the supine position. This bimodal response could be explained by a failing ANS when it is challenged and by the role of central blood volume on HRV since it increases when tilt is interrupted and blood shifts from the lower body to the cardiac chambers. Moreover, MTILT might unmask the impending ANS failure, and it should be further investigated whether HRV variables may help to detect the risk of forthcoming shock in ISCLS.

The baroreflex autonomic regulation was severely dampened in the early phases of life-threatening ISCLS flares, and this feature may contribute to the severity of shock. In physiological conditions, the heart rate, arterial pressure and respiratory oscillations exert reciprocal influences in a process known as cardiorespiratory coupling [35]. These interactions are not only mechanically mediated by the cyclic variations of the pleural pressure but are also strictly modulated by the autonomic nervous system which exerts its control through sympathetic and vagal efferences [35, 36]. Respiratory and cardiovascular neurons are localised in the same brainstem regions, particularly in the ventral medulla which contains pre-sympathetic neurons in the rostral ventrolateral medulla, premotor parasympathetic cardioinhibitory neurons in the nucleus ambiguous, and the ventral respiratory group, which includes the pre-Bötzinger complex [35, 36]. Anatomical studies of respiratory and cardiovascular neurons have demonstrated that many of these neurons have projections and axon collateral processes which extend into their neighbouring cardiorespiratory regions providing an anatomical substrate for cardiorespiratory interactions [37]. Our findings support the hypothesis that an abrupt breakdown of the autonomic control occurs during the acute phase of ISCLS, and it leaves the pressure free of baroreflex control, allowing it to oscillate entrained by respiratory activity.

The value of pressure variability has been studied in mechanically ventilated patients. There are some tools as the pulse pressure variability, measured invasively, or the plethysmographic variability index, derived non-invasively from the photo-plethysmographic wave, that has been validated to predict fluid responsiveness in patients under mechanical ventilation, but they have no value in spontaneously breathing patients. These indices are rough, just assessed in the time domain, and merely consider the mechanical heart-lung interactions. Moreover, the tachycardia observed in the acute phase does not merely mean that ANS activity is not altered. Many mechanisms induce a reflex tachycardia, both intracardiac and systemic (i.e. catecholamine incretion). What is lost during ISCLS crises is the “modulation” of ANS on the cardiovascular system, whose alteration can be detected analysing the coupling between heart rate and blood pressure oscillations. Physiologic systems are intrinsically variable [38, 39] and act as weakly coupled oscillators in the healthy state. The transition to illness is characterised by the loss of variability, or increased regularity, which in physiologic dynamics has been termed "decomplexification" [40]. In general, HRV decreases with critical illnesses, such as sepsis and multiple organ dysfunction, and returns toward normal during recovery [41]. In critically ill patients, both heart rate and arterial blood pressure variability indices showed different patterns in survivors compared to non-survivors and were correlated to APACHE-II score while “macrohaemodynamic” variables, such as mean arterial pressure, heart rate, systemic vascular resistance index, and cardiac index did not [42]. Blood pressure variability is modulated by the baroreflex control so that the cardioventilatory coupling relies more on central integrating mechanisms than on bare peripheral heart-lung interactions. Merely targeting the therapeutic interventions improvement of absolute values of vital signs (i.e. arterial pressure) does not imply a restoration of the cardiovascular autonomic control [33]. In our series, ANS appeared as a very decomplexified system in the acute phase, due to the loss of variability and baroreflex gain. The study of ANS modulation may be a window on the comprehension of the failure of central control of haemodynamics. Whether ANS assessment might be a promising tool to targeting the therapy in critically ill patients remains unknown.

This study has some limitations, the main of which is the small sample size due to the rarity of ISCLS. However, this is the first report of analyses of ANS modulation in a series of ISCLS patients during the acute, post-acute and recovery phase. Therefore, we believe that our data have a remarkable relevance, adding new insights into the knowledge of the mechanisms underlying ISCLS flares. Moreover, both sedation and positive pressure ventilation may affect ANS variables [43, 44]. However, only two cases were sedated and mechanically ventilated, and in one of them, only the acute phase recording was considered in the analysis. The second patient was sedated during all three phases considered in the analysis, thereby the possible bias of sedation is cleared because it had the same effect on each phase. Finally, it has been demonstrated that hypovolemia elicits an autonomic response in experimental settings despite anaesthesia and mechanical ventilation [45].

In conclusion, during the acute phase of ISCLS, there was an unexpected failure of the autonomic control of haemodynamics, which began to reappear during the post-acute phase and was finally restored during the recovery phase. We speculate that the remarkable derangement of HRV variables and the loss of baroreflex control might have a role as early biomarkers that characterise the forthcoming acute (fluid-unresponsive) phase and the transition to the post-acute (likely fluid-responsive) phase. Therefore, ANS indices, together with clinical indicators and a few commonly used biochemical parameters, may serve as useful tools, allowing to follow the course of ISCLS crises and helping to tailor management accordingly.

## Supporting information

S1 Dataset (RAR)
